# Hyponatremia is a surrogate marker of poor outcome in peritoneal dialysis-related peritonitis

**DOI:** 10.1186/1471-2369-15-113

**Published:** 2014-07-10

**Authors:** Min-Hua Tseng, Chih-Jen Cheng, Chih-Chien Sung, Yu-Ching Chou, Pauling Chu, Giien Shuen Chen, Shih-Hua Lin

**Affiliations:** 1 Division of Pediatric Nephrology, Department of Pediatrics, Chang Gung Memorial Hospital and Chang Gung University, Taoyuan, Taiwan; 2 Graduate Institute of Medical Sciences, National Defense Medical Center, Taipei, Taiwan; 3 School of Public Health, National Defense Medical Center, Taipei, Taiwan; 4 Division of Nephrology, Department of Medicine, Tri-Service General Hospital, National Defense Medical Center, Taipei, Taiwan

**Keywords:** Hyponatremia, Peritoneal dialysis, Peritonitis, Outcome

## Abstract

**Background:**

Hyponatremia is known to be a marker of poor prognosis in many clinical conditions. The association between hyponatremia and clinical outcomes in peritoneal dialysis-related peritonitis (PDRP) has not been studied. We evaluated the association between hyponatremia and clinical parameters of patients with PDRP.

**Methods:**

We conducted a retrospective analysis of medical records of patients with PDRP admitted to a medical center in the period 2004-2011. Patients with serum Na^+^ <130 mEq/L and ≥ 130 mEq/L at admission were divided into hyponatremic and normonatremic groups, respectively. The demographic and laboratory characteristics, pathogens of peritonitis, length of hospital stay and mortality rate were analyzed.

**Results:**

Hyponatremia occurred in 27% (27/99) patients with PDRP. Gram-negative bacilli were the major pathogen responsible for 78% (21/27) PDRP in hyponatremic group while gram-positive cocci were found in 75% (41/55) PDRP in normonatremic groups. There was no significant difference in age, duration of dialysis, PD catheter removal rate and technique failure between two groups. Hyponatremic group had significantly higher serum CRP (p <0.001), lower serum albumin (p < 0.001) and phosphate (p < 0.05). Of note, serum Na^+^ level was positively correlated with serum albumin (p < 0.001), phosphate (p < 0.04) levels, and subjective global assessment (SGA) score (p < 0.001). Moreover, the length of hospital stay was longer and in-hospital mortality rate was higher in hyponatremic group (p < 0.001). Using a multivariable logistic regression, we showed that hyponatremia at admission is an independent predictor of in-hospital mortality (OR 76.89 95% CI 3.39-1741.67, p < 0.05) and long hospital stay (OR 5.37, 95% CI 1.58- 18.19, p < 0.05).

**Conclusions:**

In uremic patients with PDRP, hyponatremia at admission associated with a high frequency of gram negative bacilli infection, low serum albumin and phosphate levels, low SGA score, and poor prognosis with long hospital stay and high mortality rate.

## Background

Hyponatremia is the most common electrolyte abnormalities in clinical medicine [1]. The estimated prevalence of hospitalized patients with serum sodium (Na^+^) less than 135 mEq/L was 15% [2,3]. Based on the underlying mechanisms, hyponatremia could stem from a surplus of electrolyte-free water (EFW) or a deficit of Na^+^ and/or K^+^. The former mechanism is rarely caused by large amount of water ingestion but the effect of ADH, which impairs free-water clearance and results in water retention [4]. Inappropriate secretion of anti-diuretic hormone (ADH) related to a variety of underlying diseases or drugs is the most common cause of euvolemic hyponatremia in patients with normal renal function. Patients with acute or chronic kidney failure are more susceptible to hypervolemic hyponatremia due to decreased renal water excretion. It has been constantly reported that hyponatremia has a significant impact on morbidity and mortality in various morbidities and also patients with chronic kidney disease, hemodialysis and peritoneal dialysis (PD) [5-7].

Continuous ambulatory peritoneal dialysis (CAPD) has been used as a popular renal replacement therapy for providing more flexibility of dialysis schedule and preserving residual kidney function. Peritoneal dialysis-related peritonitis (PDRP), the major complication of CAPD, is the leading cause of severe morbidity, technique failure and mortality in PD patients [8,9]. Although hyponatremia is not uncommon in PD patients with reported incidence up to 14.5% [10], it is still unknown whether hyponatremia associates with the clinical consequences of PDRP. Here, we investigated the impact of hyponatremia on clinical parameters and outcomes in hospitalized patients with PDRP. Results to be reported indicate that hyponatremia in patients with PDRP positively correlated with the severity of underlying peritonitis, length of hospital stay and in-hospital mortality.

## Methods

### Patients

The study protocol was approved by the Ethics Committee on Human Studies at Tri-Service General Hospital, National Defense Medical Center, in Taiwan, R.O.C. All patients provided written informed consent prior to participation. We retrospectively reviewed the medical records of unselected PD patients who admitted to a single medical center (Tri-Service General Hospital) due to PDRP from January 2004 to December 2011. These patients received CAPD with standard glucose solution for more than 3 months and were followed up monthly at CAPD clinic. All patients were on disconnect system and used 1.5 or 2 L bags of Baxter dialysate containing 1.5%, 2.5% or 4.25% glucose, which were exchanged 3-5 times per day. Dialysate sodium concentration is 132 mmol/L. A diagnosis of PDRP was made if the patient had at least two of the following criteria: (A) clinical features of peritonitis such as abdominal pain or cloudy peritoneal dialysis effluent, (B) total leukocyte count ≧ 100 cells/mm^3^, with more than 50% polymorphonuclear cells in the differential count, (C) positive gram stain or culture of peritoneal dialysis effluent. All patients with PDRP were treated with empiric antibiotics administered by the intraperitoneal route. All patients did not have hemodialysis while they underwent serum Na determination. We excluded patients who had chronic hyponatermia or hyponatremia before the event of PDRP, patients with pseudohyponatremia due to extracellular hyperosmolality secondary to hyperglycemia, hyperlipidemia or the use of icodextrin-based peritoneal dialysis or mannitol, and patients without enough clinical parameters for analysis. In addition, patients with other cormobid diseases, including hypothyroidism, adrenal insufficiency, liver cirrhosis and nephrotic syndrome, which might have impact on serum Na during hospitalization, were also excluded.

### Data collection

The following data were retrieved from the medical records of all eligible subjects: age, gender, primary renal disease, residual renal function, peritoneal membrane characteristics, dialysis adequacy, nutrition status, co-morbidity and medication. The residual renal function was measured using mean urea and creatinine clearance. The membrane transport status was evaluated by standard peritoneal equilibration test (PET) a month after initiation of CAPD and then repeated every 6 month as suggested [11]. Dialysis adequacy evaluated by dialysate volume and averaged glucose concentration and small-solute clearance determined by total Kt/V urea (the sum of peritoneal Kt/V and renal Kt/V) prior to peritonitis were recorded. We collected the laboratory data on admission, including C-reactive protein (CRP), Na^+^, K^+^, Cl^-^, creatinine, urea, total cholesterol, triglyceride, total calcium, inorganic phosphate, albumin, total protein and fasting glucose measured by standard laboratory techniques with automatic analyzer (AU 5000 chemistry analyzer; Olympus, Tokyo, Japan). The nutrition status measured as normalized protein nitrogen appearance and 7-point subjective global assessment (SGA) score was also recorded [12]. Co-morbidities, including congestive heart failure, hypertension, diabetes mellitus, liver cirrhosis, malignancy, systemic lupus erythematosus, were obtained from previous history. Anti-hypertensive agents including calcium channel blocker, α-adrenergic blocker, β-adrenergic blocker, angiotensin-converting enzyme inhibitor, angiotensin-II blocker, thiazide, spironolactone and furosemide were recorded. Microorganisms responsible for each PDRP were collected. Clinical outcomes of PDRP, including length of hospital stay, subsequent peritonitis, removal of PD catheter, failure of peritoneal dialysis and hospital mortality were evaluated.

### Definition of hyponatremia

Hyponatremia was defined as serum Na^+^ concentration <130 mEq/L for 2 consecutive measures on the first two days of admission. The patients were then divided into hyponatremic group with serum Na^+^ <130 mEq/L and normonatremic group with serum Na^+^ ≧130 mEq/L. To accurately analyze the association between serum Na^+^ and serum albumin and phosphate, the effect of hyperglycemia on serum Na^+^ concentration was adjusted by the formula as previously described [13].

### Spectrum of microorganisms

All microorganisms responsible to PDRP were recorded. To determine the influence of microbial spectrum on clinical outcome, patients with polymicrobial PDRP were excluded.

### Clinical outcomes

The clinical events and consequences including hospital mortality, length of hospital stay, removal of PD catheter, peritonitis episodes, and technique failure, were examined. Hospital mortality was defined as any death occurred during the same hospitalization or within one week after discharge. Length of hospital stay in the patients who died during hospitalization was calculated as the period from the date of admission to that of death. Peritonitis episodes were defined as any occurrence of recurrent, relapsed, and repeated peritonitis. Technique failure was defined as any failure of continued peritoneal dialysis after PDRP. To understand if hyponatremia is an independent predictor of outcomes in PDRP patients, we adjusted the effects of multiple confounders of outcome of interest including age, gender, spectrum of microorganisms and comorbidities, which was quantitatively scored by the Deyo modification of Charlson comorbidity index (Deyo-CCI) [14-16].

### Statistical analyses

Data are expressed as mean ± standard deviations for continuous variables, and percentages for categorical variables. Chi-square test was used for categorical variables and Pearson correlation coefficients for linear correlation. All of the covariates were examined in univariate analyses. Logistic regression analysis was performed to estimate the risk of increased length of hospital stay, peritonitis episodes, Technique failure, removal of PD catheter and hospital mortality. All statistical analyses were performed using SPSS for window software version 18.0 (SPSS Inc., Chicago, IL, USA). For all analyses, statistical significance was reached when a two-tailed p-value was less than 0.05.

## Results

### Patients’ characteristics

After excluding ineligible records, a total of 99 uremic patients with PDRP were enrolled for analysis. Twenty seven (27%) patients had their serum sodium <130 mEq/L in their hospital event of PDRP and were classified into hyponatremic group. The demographic characteristics are shown in Table 1. All enrolled patients did not take Na^+^ supplement before admission. While male was the majority (66%, 48/72) in normonatremic group, female was predominant (63%, 17/27) in hyponatremic group. PDRP patients who developed hyponatremia did not differ significantly in age, underlying renal diseases, nutritional condition, prescribed medication, duration of dialysis, osmolality of dialysate, duration between PDRP onset and antibiotics treatment, the burden of comorbid diseases included congestive heart failure (Deyo-CCI score: 3.6 ± 1.2 vs. 3.7 ± 1.5), and residual renal function (1.2 ± 1.4 vs. 1.5 ± 1.5 mL/min/1.73 m^2^) from those who did not.

**Table 1:** Clinical characteristics on admission of patients with PDRP

	**Hyponatremic group (n = 27)**	**Normonatremic group (n = 72)**	**p-value**
Gender (M/F)	10/17	48/24	0.015
Age (years)	49.1 ± 9.9	52.6 ± 11.7	0.173
Duration of dialysis (years)	4.0 ± 1.5	3.4 ± 1.8	0.148
Primary renal disease			
Chronic glomerulonephritis	4	15	0.696
Diabetic nephropathy	9	28	0.783
Hypertension	8	13	0.328
Systemic lupus erythematosus	3	7	1.000
Polycystic kidney disease	0	1	1.000
Unknown	3	8	1.000
Medication			
Oral Hypoglycemic agent	1	3	1.000
α-adrenergic blocker	6	8	0.197
β-adrenergic blocker	6	22	0.724
Calcium channel blocker	15	27	0.164
ACEI or ARB^+^	5	7	0.185
Furosemide	2	6	1.000
Na^+^ supplement	0	0	1.000
Number of 4.25% Dialysate (bags/day)	1.2 ± 0.8	1.0 ± 0.7	0.144
Duration before antibiotics delivery (day)	1.1 ± 0.5	1.1 ± 0.8	0.148
Co-morbidity: Deyo-CCI^++^	3.6 ± 1.2	3.7 ± 1.5	0.755
Subjective global assessment score	4.6 ± 1.2	5.7 ± 0.6	<0.001
Nutritional status: mean nPNA*	1.1 ± 0.3	1.2 ± 0.4	0.278
RRF** (mL/min/1.73 m^2^)	1.2 ± 1.4	1.5 ± 1.5	0.257

^+^ACEI or ARB, Angiorensin-convering enzyme inhibitor or Angiotensin-II receptor blocker, ^++^Deyo-CCI, Deyo-charlson comorbidity index, *mean nPNA (mg/kg/day), the 6-month average of normalized protein nitrogen appearance, **RRF, residual renal function.

### Laboratory studies and hyponatremia

As shown in Table 2, hyponatremic PDRP patients had a significantly higher serum CRP (10.9 ± 2.9 vs. 4.5 ± 1.6 mg/dL, p <0.001), lower serum albumin (2.6 ± 0.7 vs. 3.3 ± 0.5 gm/dL, p < 0.001) and lower serum phosphate (3.9 ± 1.3 vs. 5.3 ± 2.8 mg/dL, p < 0.05). The hyponatremic group tended to have lower normalized protein nitrogen appearance (nPNA), a nutritional index of PD patients (1.1 ± 0.2 vs. 1.2 ± 0.2 mg/kg/day), and SGA score (4.6 ± 1.2 vs. 5.7 ± 0.6, p < 0.001). Using multi-linear regression analysis, the serum sodium level was positively correlated with the serum albumin level (r = 0.355, P < 0.001), serum phosphate level (r = 0.205, p < 0.04), and SGA score (r = 0.352, p < 0.001). There were no significant difference in small solute clearance/U/P Cr (0.70 ± 0.1 vs. 0.64 ± 0.1), WCC (62.6 ± 10.3 vs. 68.9 ± 16.7 L/week/1.73 m^2^) and weekly Kt/V (1.9 ± 0.4 vs. 2.1 ± 0.4) and other laboratory studies between two groups.

**Table 2:** Laboratory characteristics on admission of patients with PDRP

	**Hyponatremic group (n = 27)**	**Normonatremic group (n = 72)**
Na^+^ (mmol/l)	126.8 ± 2.4*	135 ± 3.4
K^+^ (mmol/l)	3.3 ± 0.7	3.7 ± 0.8
Cl^-^ (mmol/l)	91.0 ± 2.6*	98.1 ± 4.2
Total calcium (mg/dl)	9.0 ± 1.0	9.0 ± 0.9
Phosphate (mg/dl)	3.9 ± 1.3*	5.3 ± 2.8
C-reactive protein (mg/dl)	10.9 ± 2.9**	4.5 ± 1.6
Albumin (mg/dl)	2.6 ± 0.7**	3.3 ± 0.5
Triglyceride (mg/dl)	163.9 ± 19.6	164.0 ± 24.0
Cholesterol (mg/dl)	191.3 ± 34.4	197.8 ± 20.4
Blood urea nitrogen (mg/dl)	64.3 ± 22.7	64.3 ± 21.0
Uric acid (mg/dl)	5.6 ± 1.5	6.1 ± 1.5
Glucose (mg/dl)	109.9 ± 18.5	119.7 ± 32.4
Membrane transport (U/P Cr)	0.7 0 ± 0.1	0.64 ± 0.1
WCC^+^ (L/week/1.73 m^2^)	62.6 ± 10.3	68.9 ± 16.7
nPNA^++^ (mg/kg/day)	1.1 ± 0.3	1.2 ± 0.4
Weekly Kt/V	1.9 ± 0.4	2.1 ± 0.4

*p value < 0.05, **p value < 0.001, ^+^WCC, weekly creatinine clearance, ^++^nPNA, normalized protein nitrogen appearance.

### Spectrum of microorganisms and hyponatremia

Gram-positive cocci (GPC), gram-negative bacilli (GNB) and fungus accounted for 47, 35 and 2 episodes of all PDRP, respectively. The remaining 15 episodes were culture-negative. Among patients with gram-negative peritonitis, *Pseudomonas aeruginosa* was the leading pathogen (28.6%, 10/35), followed by *Escherichia coli* (22.9%, 8/35), *Enterobacter* species (20%, 7/35), *Klebsiella pneumoniae* (11.4%, 4/35), *Acinetobacter* species (5.7%, 2/35), and the others (11.4%, 4/35). Nine of patients with gram-negative peritonitis had prodromal diarrhea at presentation. In regard to gram-positive peritonitis, the most common isolated pathogen was *Staphylococcus aureus* (48.9%, 23/47), followed by *Staphylococcus epidermidis* (29.8%, 14/47), *Streptococcus* species (12.8%, 6/47), *Enterococcus* species (6.4%, 3/47), and others (2.1%, 1/47). We compared the microbial spectrum of PDRP between two groups and showed that hyponatremic PDRP patients had higher incidence of gram-negative peritonitis (21/27, 77.8% versus 6/27, 22.2%, p <0.001), while normonatremic PDRP patients were more likely to have gram positive peritonitis (41/55, 74.5% versus 14/55, 25.5%, p < 0.05). The association analysis of microbial spectrum and clinical outcomes showed that patients with gram-negative PDRP had a significantly higher hospital mortality (6/35 versus 2/45, p = 0.022) and longer length of hospital stay (11.0 ± 4.0 versus 8.4 ± 3.2 days, p = 0.001) (Table 3). *Pseudomonas aeruginosa* was known to be highly virulent and caused higher hospital mortality rate than other pathogens (4/10, 40% versus 4/72, 5.7%). Of note, patients in this subgroup of *Pseudomonas aeruginosa*-induced PDRP had higher incidence of hyponatremia than those with non-*Pseudomonas aeruginosa* gram-negative PDRP (7/10, 70.0% versus 14/25, 56.0%).

**Table 3:** The relationship between spectrum of microorganisms and clinical outcomes

	**Length of stay**	**Technique failure**	**Removal of PD catheter**	**Peritonitis episodes**	**Hospital mortality**
GPC^++^ (47)	8.4 ± 3.2*	5	6	12	2**
GNB^+^ (35)	11.0 ± 4.0*	9	6	9	6**
Non-P.A. (25)	9.1 ± 3.6***	7	4	7	2***
P. A.^+++^ (10)	11.3 ± 4.3***	2	2	2	4***

^+^GNB, gram-negative bacilli, ^++^GPC, gram-positive cocci, ^+++^P.A., *Pseudomonas aeruginosa*, *p =0.001, **p = 0.022, ***p = 0.071, ***p = 0.003.

### Clinical outcomes and hyponatremia

**Figure 1:**
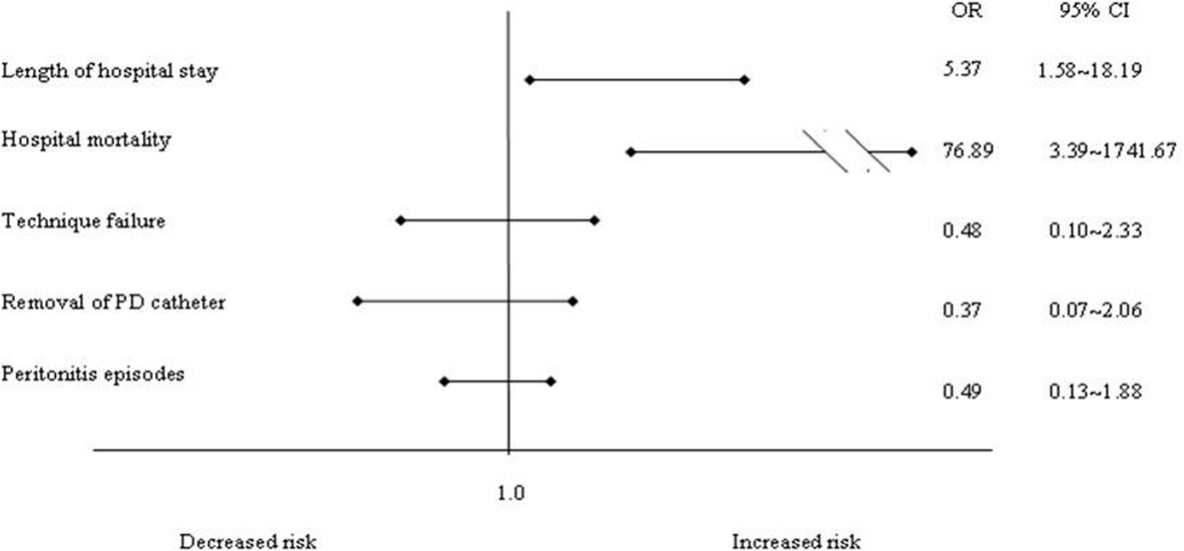
**Adjusted risk for hospital mortality, length of hospital stay, technique failure, removal of PD catheter and peritonitis episodes in patients with and without hyponatremia.** 95% confidence intervals indicating the individual contribution of hyponatremia to the respective outcomes were derived from logistic regression models and adjusted for age, gender, Deyo-CCI score and the *Pseudomonas aeruginosa* infection.

The overall hospital mortality rate of patients with PDRP was 8.1% (8/99). Hyponatremic PDRP patients had a significantly higher hospital mortality rate (25.9% vs. 1.4%, p < 0.001) than normonatremic group, even after correction of hyponatremia. Hyponatremic group also had longer lengths of hospital stay (11.9 ± 4.3 vs. 8.3 ± 2.9 days, p < 0.001) than normonatremic group (Table 4). There were no significant differences in the incidences of other hospital events, including removal of PD catheter, technique failure, and peritonitis episodes between two groups. Using univariate analysis, we identified that hyponatremia and *Pseudomonas aeruginosa* were associated with higher hospital mortality (OR 24.85 95% CI 2.89-214.02, p = 0.003; OR 14.17 95% CI 2.82-71.18, p = 0.001), and hyponatremia and female gender linked to longer length of hospital stay (OR 7.75 95% CI 2.82-21.30, p <0.001; OR 2.77 95% CI 1.10-7.03, p = 0.032) (Table 5). To eliminate the potential effects of other covariates of clinical outcomes, multivariable logistic regression were applied to adjust age, gender, Deyo-CCI score, gram negative bacilli and *Pseudomonas aeruginosa* infection, and serum Na^+^ concentration. Hyponatremia remained independently associated with increased hospital mortality (OR 76.89 95% CI 3.39-1741.67, p = 0.006) and increased length of hospital stay (OR 5.37, 95%CI 1.58- 18.19, p =0.007). However, hyponatremia did not affect the occurrence of technique failure, removal of PD catheter, and peritonitis recurrence (Figure 1).

**Table 4:** **Hospital outcomes of hyponatremia and normonatremic PDRP**^**+**^

	**Hyponatremic group (n = 27)**	**Normonatremic group (n = 72)**
Length of hospital stay	11.9 ± 4.3*	8.3 ± 2.9
Technique failure	5/27 (18.5%)	9/72 (12.5%)
Removal of PD^++^ catheter	3/27 (11.1%)	9/72 (12.5%)
Peritonitis episodes	6/27 (22.2%)	22/72(30.6%)
Hospital mortality	7/27 (25.9%)*	1/72 (1.4%)

^+^Peritoneal dialysis-related peritonitis, ^++^PD, peritoneal dialysis, *p value < 0.001.

**Table 5:** Logistic regression analysis of risk factors of hospital mortality and length of hospital stay

	**Hospital mortality**						**Length of hospital stay**^**+**^					
	**Univariate**			**Multivariate**			**Univariate**			**Multivariate**		
	**OR**	**95% CI**	**p value**	**OR**	**95% CI**	**p value**	**OR**	**95% CI**	**p value**	**OR**	**95% CI**	**p value**
Age	1.05	0.98,1.13	0.164	1.17	1.01, 1.37	0.038	0.97	0.95,1.03	0.485	1.01	0.95, 1.06	0.830
Gender^++^	0.84	0.19,3.72	0.815	0.54	0.07, 4.33	0.562	2.77	1.10,7.03	0.032	1.93	0.62, 6.06	0.259
Deyo-CCI*	1.04	0.64,1.70	0.863	0.70	0.30, 1.62	0.401	1.01	0.74,1.38	0.957	1.07	0.71, 1.63	0.737
Na**	24.85	2.89,214.02	0.003	76.89	3.39, 1741.67	0.006	7.75	2.82,21.30	<0.001	5.37	1.58, 18.19	0.007
P. A.***	14.17	2.82,71.18	0.001	5.92	0.57, 61.87	0.137	2.16	0.56,8.38	0.266	0.72	0.14, 3.74	0.698

^+^Length of hospital stay, median number of length of stay (12 days) of patients in group I was used for logistic regression analysis, ^++^Gender, female versus male, *Deyo-CCI, Deyo-charlson comorbidity index, **Na, hyponatremia versus normal ***P.A., *Pseudomonas aeruginosa*.

## Discussion

The current study represented the first to demonstrate the prognostic significance of hyponatremia in uremic patients with PDRP. Hyponatremia exerted a negative influence on length of hospital stay and in-hospital mortality independently, even after the adjustment for age, gender, spectrum of pathogens and co-morbidity. Our results suggested that hyponatremia *per se* is a surrogate marker of poor clinical outcome in uremic patients with PDRP.

The cutoff value of serum Na^+^ 130 mEq/L has been popularly used to define hyponatremia in many studies [17-19]. In this study, the incidence of hyponatremia in PDRP patients was approximately 27%, much higher than 2.4% in general hospitalization and 4.1% in patients with community-acquired pneumonia [18,19]. The reported incidences of hyponatremia in PD population varied greatly from 2.4% to 14.5% [10,17]. It seems likely that PDRP patients are more susceptible to hyponatremia than general PD patients. People have proposed several mechanisms to explain the development of hyponatremia in PD, including [1] net water gain due to excessive water intake or low ultrafiltration rate of free-water [2], negative Na^+^/K^+^ balance caused by low Na^+^/K^+^ intake or high Na^+^/K^+^ removal, and [3] shift of intracellular water to extracellular space induced by the use of icodextrin or hypercatabolism and malnutrition [17,20]. It is still unknown why PDRP patients have higher incidence of hyponatremia than other PD patients. Our speculation is that peritonitis may disrupt aquaporin-mediated water removal, reduce Na^+^ intake due to poor appetite, and, probably most important, lead to a hypercatabolic state, in which intracellular osmoles, such as protein, nucleic acids, phosphates, are degraded and thus move intracellular water into extracellular space. Compatible with this concept, we showed that the Na^+^ concentration in PDRP was positively correlated with serum albumin and phosphate levels, and SGA, indicating the relationship between serum Na^+^ and nutritional status in PDRP patients.

By analyzing the microbial spectrum of PDRP, we found that patients with hyponatremia had higher percentage of gram-negative peritonitis than those without hyponatremia. It is known that gram-negative microorganisms enter peritoneum mainly through direct transmural migration from gut [21]. The underlying pathogenesis of this phenomenon is still unclear. It is possible that the hypoalbuminemia and malnutrition (lower nPNA) cause swelling of the intestinal mucosa and loss of intestinal barrier integrity, and thus allow the normal gut flora penetrate into peritoneum [22]. Furthermore, our results demonstrated that gram-negative peritonitis, especially *Pseudomonas aeruginosa*, had poorer clinical outcome than those with gram-positive peritonitis, compatible with previous findings [23-25]. Gram-negative microorganisms have adapted to many antibiotics, especially the first-line β-lactams antibiotic treatment, and may therefore exaggerate the severity of peritonitis [26]. Similarly, Szeto et al. reviewed the gram-negative PDRP and suggested that *Pseudomonas* species were the most important cause of serious peritonitis in PD patients [27]. Although higher risk of catheter removal and technique failure were reported in patients with *Pseudomonas aeruginosa* peritonitis, we did not observe this phenomenon, probably due to small number of *Pseudomonas aeruginosa* peritonitis in this study. Culture-negative peritonitis accounted for 15.2% of all PD peritonitis in the study, similar to most of the reported series [28-30].

It seems plausible that the trend of gram-negative microorganism infection *per se* can lead to overall poorer clinical outcomes in PDRP patient with hyponatremia. However, the fact that hyponatremia associates with poor prognosis in various clinical situations implies the potential effect of hyponatremia on clinical course of PDRP. Indeed, we confirmed that hyponatremia at admission was independently associated with hospital length of stay and hospital mortality rate in PDRP patients. The underlying mechanisms of hyponatremia-induced poor clinical outcomes are mostly unknown. Therefore, it is difficult to confirm the direct causal relationship between hyponatremia and outcomes of clinical diseases at present [31]. A recent study performed microarray analysis in cells maintained in low sodium (90-127 mEq/L), and found that genes involved in cell death and survival are mostly altered [32]. These detrimental effects of hyponatremia are independent of osmolality and cause neural toxicity in vitro. In humans, hyponatremia was usually mild and may not generate the similar in vitro effects. Konstam et al had demonstrated that correction of hyponatremia by using vasopressin antagonist in patients with congestive heart failure does not improve prognosis [33]. Furthermore, Kin et al. showed that the effect of hyponatremia on mortality diminished as the severity of end-stage liver disease increased [34]. These studies support the notion that severe underlying disease worsens hyponatremia and clinical outcome and hyponatremia itself may be a surrogate marker for the severity of underlying disease [35]. Several studies have shown the association between hyponatremia and severe inflammation in clinical infectious diseases [36].

There were some limitations in this study. First, we were unable to evaluate the effect of correction of serum Na^+^ level on clinical outcome owing to the retrospective fashion of analysis. Second, although there were crude associations between hyponatremia and outcomes, we acknowledged the possibility of existence of residual confounders despite multivariable analysis. Third, this study was limited to relatively small sample size in PDRP patients with hyponatremia.

## Conclusions

Hyponatremia on admission is common in uremic patients with PDRP. It is independently associated with poor clinical outcomes including increased length of hospital stay and hospital mortality. The association of hyponatremia and poor prognosis may be a reflection of initial severity of illness. Patients with PDRP who developed hyponatremia at admission may need more aggressive therapy and more intensive monitoring during hospitalization.

## Competing interests

The authors declare that they have no competing interests.

## Authors’ contributions

MHT: Conception, design, analysis and interpretation of data, drafting the article. CCS, YCC, PC, GSC: Analysis and interpretation of data. CCC: Conception and design, and revising article. SHL: Providing intellectual input of critical importance to the work and revising the article. All authors read and approved the final manuscript.

## Authors’ information

Min-Hua Tseng first author.

## Pre-publication history

The pre-publication history for this paper can be accessed here:

http://www.biomedcentral.com/1471-2369/15/113/prepub
